# The effects of sexual selection on functional and molecular reproductive divergence during experimental evolution in seed beetles

**DOI:** 10.1093/evlett/qraf045

**Published:** 2025-11-27

**Authors:** Salomé Fromonteil, Alexandre Rêgo, Elina Immonen, Biljana Stojković, Uroš Savković, Mirko Đorđević, Johanna L Rönn, Göran Arnqvist

**Affiliations:** Animal Ecology, Department of Ecology and Genetics, Uppsala University, Uppsala, Sweden; Animal Ecology, Department of Ecology and Genetics, Uppsala University, Uppsala, Sweden; Evolutionary Biology, Department of Ecology and Genetics, Uppsala University, Uppsala, Sweden; Institute of Zoology, Faculty of Biology, University of Belgrade, Belgrade, Serbia; Department of Evolutionary Biology, Institute for Biological Research “Siniša Stanković”—National Institute of the Republic of Serbia, University of Belgrade, Belgrade, Serbia; Department of Evolutionary Biology, Institute for Biological Research “Siniša Stanković”—National Institute of the Republic of Serbia, University of Belgrade, Belgrade, Serbia; Animal Ecology, Department of Ecology and Genetics, Uppsala University, Uppsala, Sweden; Animal Ecology, Department of Ecology and Genetics, Uppsala University, Uppsala, Sweden

**Keywords:** experimental evolution, sexual selection, speciation

## Abstract

Sexual selection can be an engine of divergent evolution between closely related lineages, as a result of idiosyncratic coevolution of male and female reproductive traits. The possibility that this can contribute to speciation has ample support from comparative studies but very few experimental evolution studies have addressed the role of sexual selection in very early stages of divergent evolution. Here, we use experimental evolution to study divergent evolution between replicate lines of the seed beetle *Acanthoscelides obtectus* evolving under strong or weak sexual selection for >190 generations. We first confirm that the experimental regimes employed resulted in marked differences in the strength of sexual selection. We then indirectly assess the degree of divergent evolution of those male and female traits that affect postmating sexual selection, by crossing replicate lines. We find that lines evolving under strong sexual selection are more divergent in reproductive traits, as evidenced by a stronger male × female interaction for male sperm competition success. Finally, we assess the degree of divergent evolution in the expression of candidate genes for male seminal fluid proteins and female reproductive proteins. We find that lines evolving under strong sexual selection are more divergent in the expression of reproductive proteins, providing a possible causal mechanism contributing to the results seen in the reproductive phenotype. Our findings provide evidence for more divergent evolution of reproductive traits under stronger sexual selection, in line with the tenet that sexual selection may promote divergence even in the absence of environmental differences between populations.

## Introduction

Many distinct processes can render different populations of a species to evolve toward reproductive isolation (Schluter, 2000), but determining their relative importance has proven to be difficult (Schluter, 2009; Servedio & Boughman, 2017). Several related facts strongly suggest that sexual selection often plays a key role in divergent evolution and speciation on longer time scales (e.g., Mendelson & Safran, 2021; Panhuis et al., 2001; Ritchie, 2007; Snook et al., 2009; West-Eberhard, 1983). First, by acting directly on reproductive traits, sexual selection should be particularly likely to affect reproduction between diverging lineages. Second, divergent populations or closely related species generally do tend to be more different from one another in sexual traits (e.g., Arnqvist, 1998; Cooney et al., 2019) and reproductive genes (e.g., Rowe et al., 2020; Swanson et al., 2001; Walters & Harrison, 2010), which are the targets of sexual selection, than in other types of traits and genes. Third, lineages showing signs of stronger sexual selection show relatively higher species richness in several groups (e.g., Arnqvist et al., 2000; Janicke et al., 2018, 2025; Owens et al., 1999). Fourth, male–female coevolution driven by sexual selection is predicted to be idiosyncratic and generate divergence, even in the absence of environmental differences between populations (Arnqvist & Rowe, 2005; Lande, 1981; Rice et al., 2005; West-Eberhard, 1983). For example, as a result of mutation-order effects (Mendelson et al., 2014; Rundle & Rowe, 2018), different lineages are expected to show evolutionary modification in arbitrary and unique sexual traits and reproductive genes, which may contribute to functional reproductive divergence between lineages.

A potentially important arena for divergent evolution in animals with internal fertilization is formed by reproductive proteins that affect postmating processes and male sperm competition success (Howard, 1999; Howard et al., 2009). In brief, males transfer a great number of bioactive seminal fluid proteins (SFPs) to females at mating that are important determinants of female reproduction and male fertilization success (Avila et al., 2011; Larson et al., 2012; Perry et al., 2013; Wigby et al., 2020). Females, in turn, express a multitude of female reproductive proteins (FRPs) that influence how they respond to SFPs (Gasparini et al., 2020; McCullough et al., 2020; Plakke et al., 2019; Veltsos et al., 2022; Yapici et al., 2008). Male SFPs often evolve rapidly as a class and show hallmarks of positive selection (Andrés et al., 2006; Findlay et al., 2008;Wong et al., 2008 ) and this is believed to be the result of male–female coevolution involving SFPs and FRPs (Chapman, 2018; Gasparini et al., 2020; Hollis et al., 2019; Manier et al., 2013; Sirot et al., 2015). Populations may thus diverge via postmating sexual selection that drives male–female coevolution in arbitrary and different directions in a highly multidimensional space formed by sex-specific reproductive proteins (Andrés & Arnqvist, 2001), and this may eventually result in reproductive incompatibility (Price, 1997).

Although comparative studies have provided support for a role for sexual selection in divergent evolution (Janicke et al., 2025), the comparative approach has limitations as it captures the outcomes after divergence has already occurred. By studying the very early stages of divergence, experimental studies can potentially identify a role for selection prior to the evolution of actual reproductive incompatibilities (Via, 2009). Yet, few experimental evolution studies have directly assessed such a role (Castillo et al., 2015; Edward et al., 2010; Fry, 2009; Martin & Hosken, 2003; Syed et al., 2017; Wiberg et al., 2021). We employ long-term experimental evolution in replicate lines of the seed beetle *Acanthoscelides obtectus* experiencing evolutionary regimes expected to show relatively weak (WSS) or strong sexual selection (SSS) to test whether replicated populations show elevated functional and transcriptomic divergence under stronger sexual selection. Seed beetle males transfer a range of different proteins to females at mating (Bayram et al., 2017, 2019) and females express a number of proteins in their reproductive tract after mating (Bayram et al., 2019). Further, since female seed beetles mate multiply, postmating sexual selection is pronounced (Brown & Eady, 2001; Fritzsche & Arnqvist, 2013; Hotzy & Arnqvist, 2009; Wilson et al., 1997) and the composition of the blend of SFPs in the ejaculate is correlated with male competitive fertilization success (Goenaga et al., 2015; Yamane et al., 2015). Some SFPs are beneficial for females (Savalli & Fox, 1999; Takakura, 2006; Yamane et al., 2015), while others have toxic effects (Das et al., 1980; Yamane, 2013). A recent comparative genomic study showed that SFPs evolve divergently and showed signs of correlated evolution with FRPs in this group of beetles (Papachristos et al., 2024). Here, we first assess whether sexual selection is indeed weaker in WSS lines than in SSS lines. We then test the prediction that replicate SSS lines should have diverged further from one another than WSS lines in male and female in reproductive traits, by functional assays of the outcome of sperm competition. Finally, we assess divergence between lines in the expression of candidate reproductive genes, including both SFPs and FRPs.

## Materials and methods

### Experimental evolution lines

A base population of *A. obtectus*, from which all lines were originally derived, was established in 1983 by mass mating equal numbers of adults from three local subpopulations of *A. obtectus* collected near Belgrade (Tucić et al., 1996). The base population was maintained at a large size (*N* ≈ 5,000 individuals) on *Phaseolus vulgaris* seeds in the laboratory for 3 years (27 generations), prior to founding the replicated lines. The lines were initiated and subsequently reared under either of two divergent evolutionary life history regimes, and we here refer to (Tucić et al. 1996, 1997) for a more detailed description of these. Briefly, in one regime (*N* = 4 lines; here WSS), adult beetles were allowed to mate and reproduce only during days 1–2 of adult life. Because females show a postmating refractory period of 1–2 days, females in WSS lines should be largely monandrous, which should render sperm competition rare and markedly reduce the strength of sexual selection (Šešlija et al., 2008, 2009). In the other regime (*N* = 4 lines; here SSS), both males and females were allowed to compete for matings and fertilizations from day 1 of adult life until death, but females were allowed to lay eggs only from day 10 of adult life. Here, both sexes thus mate multiply, which introduces sperm competition and should result in a stronger sexual selection regime in SSS lines (Šešlija et al., 2008, 2009; Stojković et al., 2011). Earlier work with these lines has shown that remating rates are considerably higher in SSS lines relative to WSS lines (Šešlija et al., 2008, 2009). However, while there was no evidence for pre-mating sexual isolation across replicate lines in either regime, this earlier work found that mate choice favored individuals from the own population in the WSS but not the SSS regime, possibly as a side effect of lower mating speed in WSS lines (Stojković et al., 2011). The census population size (number of potentially reproducing individuals) has been kept at approximately *N* ≈ 400 individuals per line in both regimes, following the startup phase of these lines. We maintained the lines on *P. vulgaris* seeds in an incubator under adult aphagy, at 29 °C, 55% relative humidity (RH) and a 12L/12D light regime. The two sexual selection assays described below used beetles from generation 341/225 (WSS/SSS; because of inevitable difference in generation time), which were maintained under standard common garden conditions for more than five generations prior to the assays to exclude any parental environmental effects. The RNA sequencing (see below) was done in generation 282/193, again following more than five generations of common garden conditions.

### Sexual selection assays

**Figure 1.**
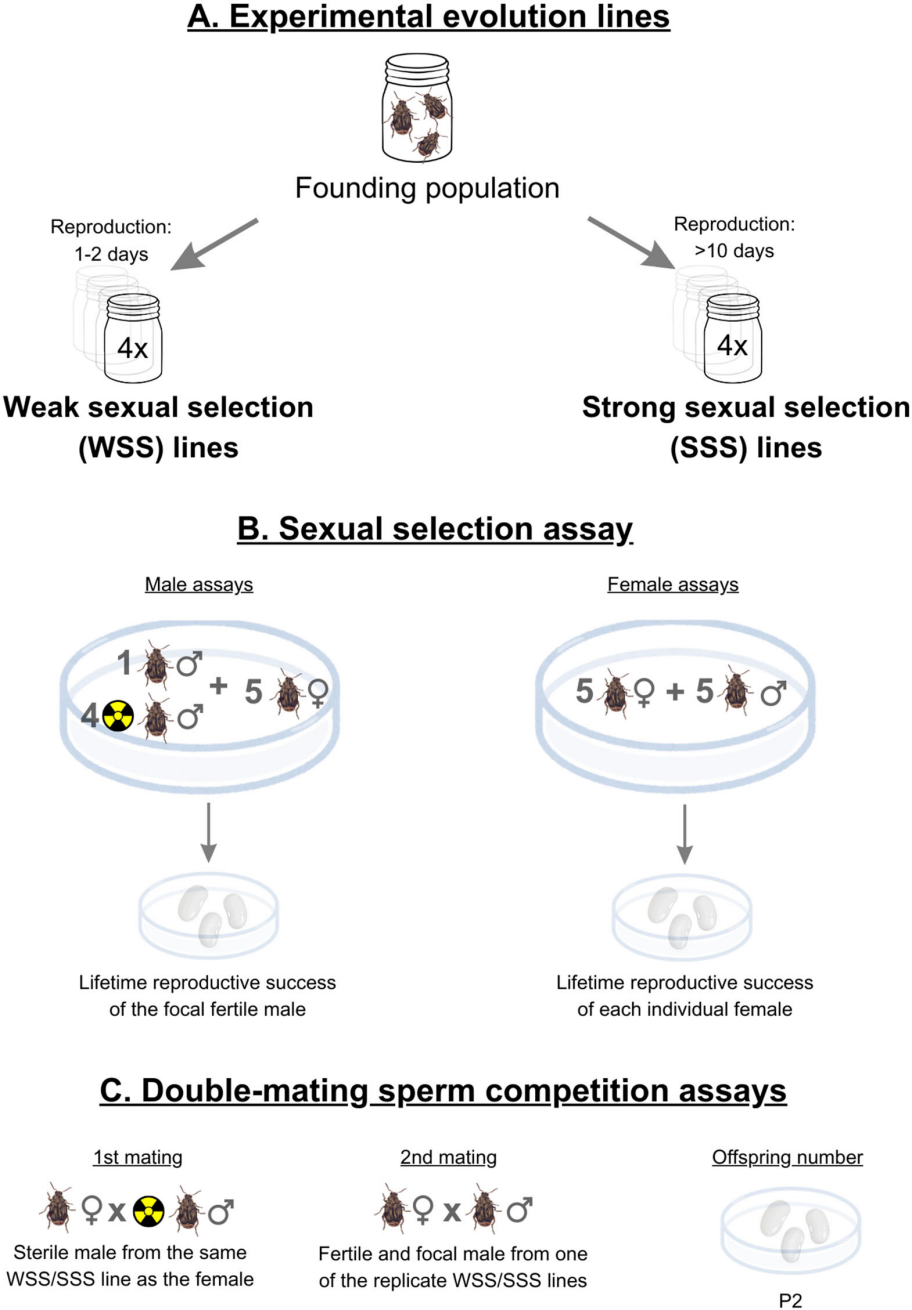
Schematic representation of the experimental design. (A) We employed replicated experimental evolution lines evolving under divergent reproductive regimes, that should result in weak or strong sexual selection, for >190 generations. (B) Sex-specific competitive reproductive assays were performed to estimate the strength of sexual selection in the two regimes. (C) Finally, divergence between lines in postmating fertilization success was estimated in a series of standard double-mating experiments, involving first males sterilized by irradiation. See text for further details. Artwork by M. Trabert.

We first characterized sexual selection in the WSS and SSS experimental evolution regimes quantitatively, by employing the experimental design previously used by Fritzsche and Arnqvist (2013) in several other seed beetle species (Figure 1). Briefly, 1-day post-emergence virgin males and females were collected from each of the eight replicate experimental evolution lines and were individually color marked on their elytra. We then scored individual mating and reproductive success across many replicated “mating populations,” each consisting of five males and five females introduced into a mating dish, using a design similar to that of Bjork and Pitnick (2006). We estimated sexual selection independently for males and females, using separate male (*N* = 5 mating populations per replicate line) and female (on average *N* = 2.5 mating populations per replicate line) assays. In the male assays, four randomly selected males in each mating population were made sterile for life by irradiation (X-ray; dose = 70–80 Gy) while a single focal randomly selected male was a normal fertile male. Sperm cells of irradiated males are fully motile and are able to successfully compete for fertilizations, but eggs fertilized by sperm of irradiated males do not hatch after laying (Harano et al., 2007; Maklakov & Arnqvist, 2009). In the female assays, in contrast, all males were normal and fertile. Each replicate mating population thus yielded data on the mating and reproductive success of a single male in the male assays, while it yielded the same data for five females in the female assays.

In each mating population, males and females were allowed to mate freely for 1 hr each day, in 90-mm Ø petri dishes at 30 °C. All beetles were continuously monitored and we recorded the total number of copulations per individual by direct observation. We scored male–female interactions as a copulation if the male adopted the typical mating position, where his forelegs no longer rested on the female’s back and the aedeagus was inserted, for at least 1 min (Düngelhoef & Schmitt, 2006). Between these mating episodes, all females were isolated individually in petri dishes in climate chambers. To mimic reproductive conditions in the experimental evolution regimes, dishes for females from assays involving WSS lines were provided with an *ad libitum* supply of beans for oviposition from day 1 and these assays were terminated (females removed from dishes) at day 3. Dishes for females from assays involving SSS lines were not provided with beans until day 10 of adult life and these assays continued until death. Beans were subsequently incubated in climate chambers (30 °C and 55% RH) until all offspring had hatched. In the female assays, lifetime reproductive success of an individual female was measured as the total number of offspring she produced. In the male assays, lifetime reproductive success of the focal fertile male was measured as the total number of offspring produced by all five females in his mating population.

We then estimated three related measures of the strength of sexual selection in WSS and SSS lines (Jones, 2009). Relative measures were first gained by dividing metrics with sex-specific means for each experimental evolution regime. Relativizing data per mating population instead yielded results quantitatively similar and qualitatively identical to those reported here. (1) The opportunity for sexual selection (*I*_s_) was estimated as the variance in relative mating success. (2) The Bateman gradient (*β*_B_) was estimated as the slope of a regression of relative reproductive success on absolute mating success, here estimated in linear mixed models including replicate line as a random effect. These estimates are thus based on the assumption that replicate lines within an experimental evolution regime share a common Bateman gradient. (3) The Jones index (*s’*_max_) estimates the maximum strength of sexual selection, and was estimated as the product between the square root of *I*_s_ and the slope of the least-squares regression of relative reproductive success on relative mating success (*s’*_max_ = √*I*_s_ × *β*_ss_).

A few individuals with zero mating and reproductive success (*N* = 3 males [*N* = 1 WSS and *N* = 2 SSS] and *N* = 12 [*N* = 10 WSS and *N* = 2 SSS] females in total) were excluded from these computations, for two reasons. First, all copulations were recorded in our assays, so the fact that individuals with zero mating success also had zero reproductive success requires no estimation and including such zero individuals can lead to bias (Mobley & Jones, 2013). Second, and more importantly, we were interested in estimating whether and how reproductive fitness accumulates over successive matings, rather than the component of sexual selection, which derives from zero mating success resulting from a lack of suitable partners (Rhainds, 2010), as this is likely to be very low under the experimental evolution conditions. The estimation of sexual selection was thus based on the lifetime mating and reproductive success of in total *N* = 36 individual males and *N* = 88 individual females.

### Sperm competition assays

To assess functional divergence across replicate experimental evolution lines within the WSS and SSS regimes, we conducted a series of standard double-mating sperm competition assays using the sterile male technique (see Simmons, 2001). Replicate females from all lines were first mated with a sterile reference male from their own line and then with a second focal fertile male from one of the four lines within their own experimental evolution regime. The proportion of eggs laid after the second mating that resulted in offspring (i.e., P2) provides our measure of sperm competition success of focal males.

One-day post-emergence virgin females were collected and each introduced with a virgin male made sterile by irradiation (X-ray; dose = 70–80 Gy) from their own respective replicate line, into a 6-cm Ø petri dish for 1 hr. Males were removed following copulation. Irradiated males copulate and transfer sperm in a normal fashion, but eggs fertilized by irradiated sperm are inviable (Maklakov & Arnqvist, 2009). Following their first mating, females were then allowed to mate a second time after 24 hr using the same protocol but this time with a focal virgin fertile male selected randomly from one of the four replicate lines within their own regime (resulting in 4 × 4 + 4 × 4 = 32 cells in the design). All copulations were confirmed by visual observation (see above). Females that mated a second time were then transferred to a 90-mm Ø petri dish provided with an *ad libitum* supply of *P. vulgaris* beans, placed under rearing conditions. The total number of eggs laid was subsequently (following female death) counted and the total number of offspring hatched from each female, i.e., those fathered by the second fertile male, was recorded. Egg-to-adult survival is very high in seed beetles under these conditions (>90%; e.g., Rankin & Arnqvist, 2008) and we thus used the proportion of number of hatched offspring over total number of eggs laid as our measure of male sperm competition success (i.e., P2). We included only females where ejaculates from two males demonstrably competed over fertilizations, thus excluding females that did not mate during their first or second opportunity and those where the focal male failed to successfully transfer an ejaculate (i.e., P2 = 0; Simmons, 2001). The total sample size was *N* = 225 females (on average, *N* = 7 females per cell).

Variance in P2 was analyzed in generalized linear models of the number of offspring hatched, using binomial errors with the total number of eggs laid as the binomial denominator, a logit link function and an empirically derived dispersion parameter to account for overdispersion. Our design was fully crossed within each regime and models were thus fitted separately for WSS and SSS, using female line, focal male line, and their interaction as fixed effect factors. We make two specific predictions. First, P2 should overall be higher in SSS lines, as a result of relaxed selection for sperm competition success in WSS lines. Second, and more importantly, the male × female interaction effect should be stronger among SSS lines (Lüpold et al., 2020), as a result of more divergent male–female coevolution of sex-specific traits that affect the reproductive outcome of sperm competition.

### Divergence in gene expression

To compare divergence in gene expression among experimental evolution lines, we utilized the read count data from Immonen et al. (2023) (NCBI SRA, BioProject accession PRJNA492259), obtained from the reproductive tissues (i.e., the abdomen). Briefly, virgin male and female beetles were collected from the SSS and WSS lines and following standard RNA extraction and library preparation, libraries (2 sexes × 2 regimes × 4 replicate lines) were sequenced on four lanes using the Illumina HiSeq2500 system, paired-end 125-bp reads and v4 sequencing chemistry. Following a genome-guided assembly and functional annotation of the transcriptome (see Immonen et al., 2023), all read count data were analyzed using DESeq2 (Love et al., 2014) in R (R Core Team, 2021). All read counts were normalized by a variance stabilizing transformation prior to downstream analyses.

Reproductive proteins have not been annotated in *A. obtectus* and we therefore leaned on the experimental functional annotation of such proteins in the related seed beetle *Callosobruchus maculatus*, where proteins from the female reproductive tract and male seminal fluid have been identified in extensive experiments involving stable isotope labeling in combination with targeted proteomic analyses of the reproductive organs (Bayram et al., 2019). Lists of orthologs of *C. maculatus* in *A. obtectus* were derived by extracting *C. maculatus* protein sequences for 317 SFPs and 231 FRPs from this previously identified set (Bayram et al., 2019). We then performed tBLASTn using the rBLAST package (Hahlser & Nagar, 2024), querying each *C. maculatus* protein sequence against the full transcriptome of *A. obtectus* (Immonen et al., 2023). High-quality orthologous matches were identified by filtering results to retain only those with ≥90% sequence identity. For each query protein, we selected the single best match based on the lowest e-value to ensure unique ortholog assignments. This approach successfully identified 141 FRP orthologs and 72 SFP orthologs from our initial set of *C. maculatus* proteins, providing the basis for subsequent comparative expression analysis between sexual selection treatments. We then compared variation in expression between four sets of genes, defining transcripts as expressed when a raw read of >2 cpm was observed across all compared samples: (1) all orthologs of FRPs expressed in the female samples (*N* = 96), (2) all orthologs of SFPs expressed in the male samples (*N* = 50), (3) all transcripts expressed in the female samples (*N* = 18,024), and (4) all transcripts expressed in the male samples (*N* = 18,850).

To estimate divergent evolution of gene expression among replicate lines evolving under SSS relative to those evolving under WSS, we calculated divergence ratios. For each protein, we first calculated the mean expression among the four replicates of each regime. The average of the absolute difference in expression between replicates and their regime-specific mean was then used a measure of overall divergence between replicates within a regime. Divergence among SSS lines was then divided with that among WSS lines, to yield a divergence ratio expected to equal unity if evolution of gene expression was equally divergent among SSS as among WSS lines. We then asked whether the distribution of divergence ratios of reproductive proteins is consistent with that of all transcripts of the corresponding sex, using Kolmogorov–Smirnov (KS) tests comparing set 1 with 3 and 2 with 4. If found to differ, we used Mann–Whitney (MW) *U* tests to test the prediction that reproductive proteins should show greater ratios of divergence than the genome-wide background. Statistical analyses were performed with R (R Core Team, 2021) and Genstat v.18.1.0.17005.

## Results

### Sexual selection

**Figure 2.**
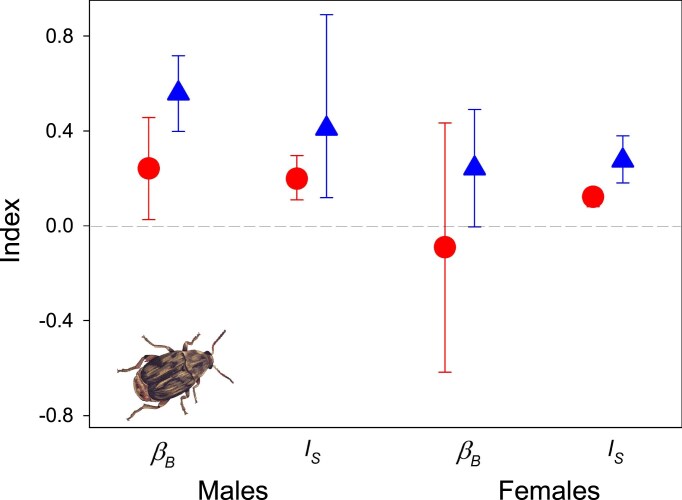
Estimates of sex-specific indices of sexual selection in lines under weak (circles) and strong (triangles) sexual selection. Given are Bateman gradients (*β*_B_) and the opportunity for sexual selection (*I*_s_). Error bars represent 95% CIs (parametric CI for *β*_B_ and bootstrap CI for *I*_s_). See text for statistical evaluation. Artwork by M. Trabert.

The mean number of lifetime matings across all females was 1.0 (*SD* = 0.67) in WSS lines and 2.1 (*SD* = 1.16) in SSS lines, confirming that the former are more monandrous and the latter more polyandrous (Šešlija et al., 2008, 2009). In males, both the opportunity for sexual selection (*I*_s_) and the Bateman gradient (*β*_B_) were about twice as high in SSS compared to WSS lines (Figure 2), and the Bateman gradient significantly so (test of equal slopes: *Z* = 2.49, *P* = 0.013) (Clogg et al., 1995). This is also reflected by the Jones index (*s’*_max_), which was three times higher in SSS lines (1.29 vs. 0.43). In females, sexual selection was overall weaker than in males in both selection regimes, as is typical in seed beetles with conventional sex roles (Fritzsche & Arnqvist, 2013; Martinossi-Allibert et al., 2020). Although sexual selection in females did tend to be higher in SSS than in WSS lines (Figure 2), the Bateman gradient did not differ significantly in the two selection regimes in females (*Z* = 1.159, *p* = 0.246). The female Jones index was, however, considerably higher in SSS (0.28) than in WSS (−0.04) lines.

These analyses verify that, as expected, postmating sexual selection is more pronounced and sexual selection two to three times stronger under SSS conditions in males. Under WSS conditions, females appear not to generally gain from mating more than once and most females were also monandrous. Under SSS conditions, the female Bateman gradient was positive, albeit marginally nonsignificantly different from zero (*t* = 1.81, *p* = 0.076), suggesting that remating is beneficial and most SSS females were also polyandrous.

### Sperm competition

**Figure 3.**
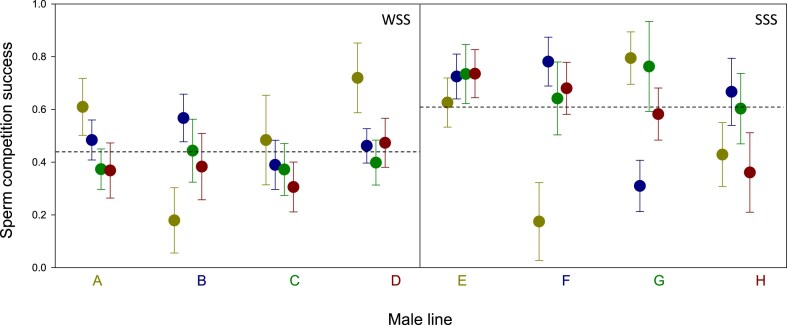
Sperm competition success of the second male to mate (P2) in double mating experiments in lines evolving under relatively weak (left) and strong (right) sexual selection. Colored letters along the abscissa denote male line identity and female line identity is given by the matching color of the circle (e.g., red circles in the left plot are females from line D). Error bars represent standard errors. Dashed lines represent mean sperm competition success in the two experimental evolution regimes.

Across all line-specific combinations, predicted mean P2 from the models given in Table 1 was overall higher within the SSS regime (predicted mean P2 = 0.61; 95% CI: 0.55–0.66) than it was within the WSS regime (predicted mean P2 = 0.44; 95% CI: 0.39–0.49). The difference in overall P2 between WSS and SSS lines was also significant both when comparing mean male line-specific P2 (i.e., across all matings males of a given line participated in) (separate variance *t*-test: *t* = 3.70, df = 4.58, *p* = 0.016, bootstrap *p* = 0.039) and when instead comparing mean female line-specific P2 (*t* = 3.32, df = 5.96, *p* = 0.016, bootstrap *p* = 0.034) (Figure 3).

**Table 1.** Generalized linear models of the fixed effects of male and female genotype (i.e., line identity) on the sperm competition success of the second male to mate a female (P2), within the two experimental evolution regimes.

EE regime	Effect	df	Deviance	*F*	*p*
WSS	Male line	3	16.01	0.75	0.528
	Female line	3	30.47	1.42	0.242
	Male line × female line	9	65.52	1.02	0.432
	Residual	97	694.49		
SSS	Male line	3	96.26	2.06	0.111
	Female line	3	19.86	0.43	0.735
	Male line × female line	9	340.99	2.43	0.015
	Residual	96	1,495.35		

*Note*. EE = experimental evolution; SSS = strong sexual selection; WSS = weak sexual selection.

**Figure 4.**
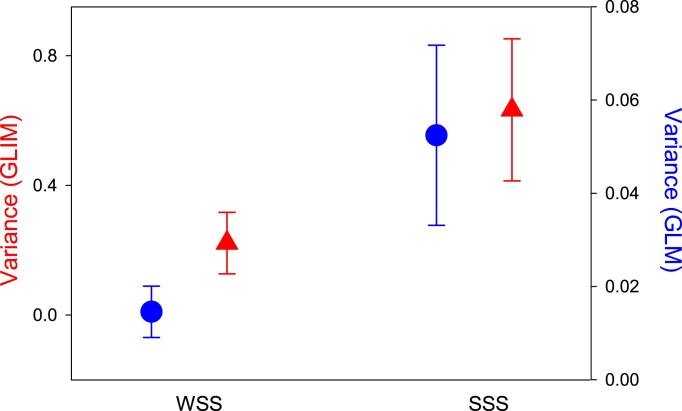
Variance component estimates of the male line × female line interaction effect from generalized (GLIM, red) and general (GLM, blue) linear mixed models of variance in last male sperm competition success, across lines with weak (WSS) or strong (SSS) sexual selection. Error bars represent standard errors.

Further, our models showed no significant male × female interaction among WSS lines (*p* = 0.432) while this interaction was significant and sizeable among SSS lines (*p* = 0.015). The effect size of the interaction term was more than twice as large among SSS (*η^2^* = 0.19) than among WSS (*η^2^* = 0.09) lines in our main models (Table 1). However, the fact that the interaction term was larger and significant among SSS lines but not significant among WSS lines does not in itself show that the importance of the male × female interaction is significantly different in the two sets of lines. Testing our second prediction regarding the relative effect of the interaction term is not trivial (Bacigalupe et al., 2007), but meaningful comparison can be made. Here, we used three complementary inferential routes. First, we fitted generalized linear mixed models with a fixed dispersion parameter, analogous to those in Table 1 but instead treating the intercept as fixed and line as random effects, and extracted the estimated focal sample variance component and its standard error for the random male × female interaction term. This was almost three times as high among the SSS lines (0.633; *SE* = 0.219) compared to the WSS lines (0.222; *SE* = 0.095). Second, we fitted general linear mixed models, using arcsine square root transformed P2 as the response and weighted by total egg count, analogous to those above (intercept as fixed and line as random effects). This allowed a variance ratio test of the variance accounted for by the male × female interaction, which was again found to be higher in the SSS lines (*F*_9,9_ = 5.29, *p* = 0.010). The interaction term in WSS lines accounted for 7.8% and in SSS lines 19.8% of total variance in arcsine square root transformed P2. Third, we fitted models analogous to those in Table 1 but without interaction terms, and extracted the residual number of offspring fathered by each male (i.e., observed—predicted). The mean residual per cell in our design (16 cells in each regime) then measures the deviation from the additive expectation (i.e., when not accounting for interaction effects). Bootstrap resampling (9,999 bootstraps) of the variance among these mean residuals across the 16 cells showed that the magnitude of the overall deviation from the additive expectation was several times higher in the SSS regime (average variance: 38.6; 95% BCa CI: 9.12–63.63) compared to the WSS regime (average variance: 6.2; 95% BCa CI: 0.77–12.91). Further, the estimated variance of this deviation was significantly higher among SSS compared to among WSS lines (variance ratio test: *F*_15,15_ = 6.2, *p* < 0.001; Bartlett’s test for homogeneity of variances: χ^2^_1_ = 10.7, *p* = 0.001), showing a larger influence of sources other than additive effects in SSS lines. These analyses thus all support the conclusion that the specific combination of male and female genotype (i.e., line identity) had a greater impact on P2 among SSS lines than among WSS lines (Figure 4). Finally, we note that within-line crosses did not generally have a significantly higher P2 than between-line crosses, either in the WSS (rank sum tests: *p* > 0.13) or the SSS (*p* > 0.73) regime, nor in both regimes combined (*p* > 0.31).

### Divergence in gene expression

**Figure 5.**
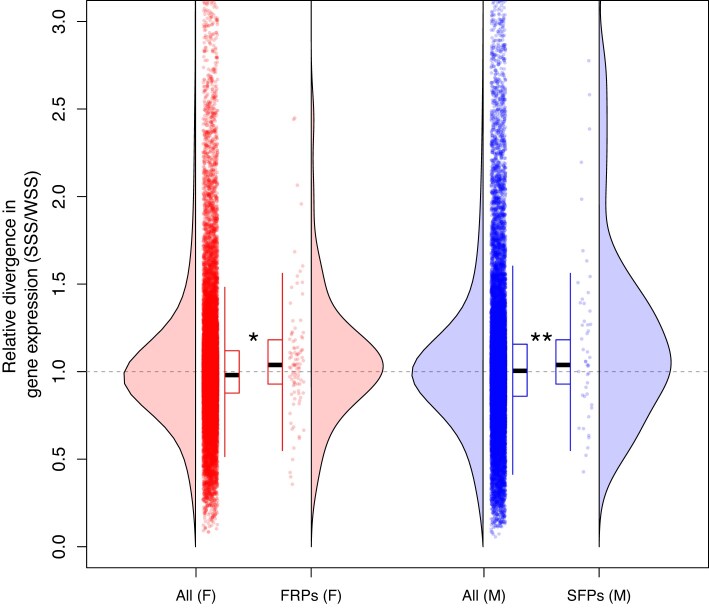
The distribution of divergence ratios for different classes of proteins. Shown from left to right are all genes expressed in females, female reproductive proteins expressed in females, all genes expressed in males, and male seminal fluid proteins expressed in males. Dashed gray line indicates equal divergence between replicate lines under strong as under weak sexual selection, larger values represent higher divergence under strong sexual selection. Note that a small fraction of proteins falls outside of the range of the ordinate used here, for illustrative purposes. For each set of genes, the distribution and underlying raw data are presented along with a corresponding boxplot indicating the interquartile range (box), 1.5 × IQR (tails), and the median (black bar). See text for statistical evaluation.

Transcriptome-wide divergence ratios were close to unity across all expressed genes in both males and females (mean bootstrap [9,999 replicates] median; males = 1.0044 [95% BCa CI: 1.0013–1.0075], females = 0.9802 [95% BCa CI: 0.9778–0.9830]), despite the fact that SSS lines by necessity have experienced fewer generations of experimental evolution. However, divergence ratios of reproductive genes were overall higher than unity for reproductive proteins, especially so for SFPs (mean bias corrected [BC] bootstrap [9,999 replicates] median; SFPs = 1.0997 [95% BCa CI: 1.0157–1.2703], FRPs = 1.0359 [95% BCa CI: 0.9970–1.0849]) (Figure 5). Moreover, we found that the distribution of divergence ratios for reproductive proteins was significantly different from that of all proteins, both for SFPs in males (KS test, *D* = 0.643, *p* < 0.001) and for FRPs in females (KS test, *D* = 0.197, *p* = 0.001). Although divergence ratios were variable across specific proteins, average divergence ratios were significantly larger for both types of reproductive proteins compared to the reference sets (MW tests: SFPs, *p* = 0.004; FRPs, *p* = 0.012) (Figure 5). These analyses thus show that the expression of reproductive proteins is on average more different between replicate SSS lines than between replicate WSS lines, and this difference in divergent evolution was significantly greater among reproductive proteins than among proteins in general.

## Discussion

A central prediction of sexual selection theory is that lineages under strong sexual selection should show more divergent evolution of reproductive traits, even in the absence of ecological differentiation, and this should be especially true for complex or polygenic traits (Mendelson & Safran, 2021; Mendelson et al., 2014; Rundle & Rowe, 2018). This prediction has comparative support (e.g., Arnqvist et al., 2000; Janicke et al., 2018, 2025; Owens et al., 1999), but support from evolutionary studies experimentally manipulating proxies of sexual selection is more limited (Plesnar-Bielak et al., 2013; White et al., 2020). This may in part be because most studies assess divergence between treatments (e.g., Hollis et al., 2019; Wiberg et al., 2021) rather than between replicate lines within treatments, but also because they often focus on pre-mating reproductive isolation (e.g., Bacigalupe et al., 2007; Wigby & Chapman, 2006), which may only emerge over time scales longer than those feasible in experimental evolution studies (Fry, 2009). Martin and Hosken (2003) found that replicated larger dung fly populations with more sexual conflict diverged to a greater degree, in traits apparently affecting successful copulations, but assessing the role of sexual selection on divergence here was complicated by potentially confounding effects of marked differences in effective population size (Tregenza, 2003; Wigby & Chapman, 2006). Using replicated lines of fruit flies evolving under male- or female-biased sex ratios, Syed et al. (2017) were able to document a larger divergence among male-biased lines in the outcome of sperm competition, consistent with a role for sexual selection in promoting early divergence.

Here, we first confirmed that our SSS lines, evolving under a regime where sexual selection should be more intense, indeed experienced an elevated incidence of postmating sexual selection and stronger sexual selection overall, particularly in males. We found that experimental populations evolving under a selective regime involving sperm competition showed a higher degree of last male sperm precedence, which aligns well with several studies showing that sperm competition promotes the evolution of traits that increase male paternity success (e.g., Firman & Simmons, 2011; Hollis et al., 2019; Hosken et al., 2001; Nandy et al., 2013; Simmons & García-González, 2008). More importantly, our functional sperm competition assays showed that males and females evolving under stronger sexual selection had diverged more in whatever male and female traits determine last male sperm competition success, relative to those evolving under weaker sexual selection. This was evident from the stronger interaction effect across SSS than across WSS lines in terms of the outcome of sperm competition (Lüpold et al., 2020).

Our result reverberates that of Syed et al. (2017), and is consistent with the view that early divergence between populations in reproductive traits is indeed promoted by sexual selection leading different populations into distinct evolutionary paths in a multidimensional reproductive trait space. Here, key candidate traits are sex-specific reproductive proteins involved in determining the outcome of postmating events and we assessed divergence in the expression of candidate genes encoding such proteins. In line with our predictions, we found that the expression profiles of candidate genes encoding both FRPs and SFPs were more divergent across replicate lines evolving under an SSS than under a WSS regime. This difference was greater among reproductive proteins than among proteins in the reference gene set, which quantifies the transcriptome-wide level of expression divergence between lines (Figure 5). While this result is novel and consistent with a role for FRPs and SFPs in determining the divergent outcome of sperm competition, we suggest that it should be interpreted as tentative as there are a few important caveats. First, it is far from obvious what a relevant reference set of proteins should be to benchmark the evolution of expression of reproductive proteins, as they are somewhat unusual in several ways. For example, most are secreted proteins and many show sex-biased expression, both of which could potentially affect the rate of evolution of their expression (Dapper & Wade, 2020; Winter et al., 2004). Second, our gene set involving reproductive genes is most likely not a complete representation of the entire reproductive proteome of *A. obtectus*. Their functional annotation (Bayram et al., 2019) was performed in another species (estimated divergence time > 45 Myo) and not all proteins were found to be expressed in our *A. obtectus* lines. The latter is likely due to the rapid evolution of reproductive proteins in this group (Bayram et al., 2017; Papachristos et al., 2024) but could also be influenced by the fact that we used bulk RNA from the abdomen, which limits detection of rare transcripts. Ideally, the comparison should have been based on a dedicated experimental proteomic annotation of reproductive proteins in *A. obtectus* and on more tissue-specific samples of RNA.

Experimental evolution studies of the effects of sexual selection typically employ manipulations of operational sex ratios or “remove” sexual selection by enforcing monogamy (Edward et al., 2010; Hollis et al., 2019). In reality, however, it is very difficult indeed to entirely isolate the effects of sexual selection simply because any modification of the social setting will not only affect the opportunity for sexual selection, but will also have potential direct or indirect effects on natural selection or demography (e.g., Edward et al., 2010; Whitlock & Agrawal, 2009; Wigby & Chapman, 2006). This is true also in our case (Stojković et al., 2011), which forms an additional caveat. While we show here that sexual selection has indeed been stronger in our SSS lines, there are reasons to believe that natural selection may also have been stronger (Stojković et al., 2011; Tucić et al., 1996). Although it is not obvious that traits involved in determining the outcome of sperm competition, such as SFPs and FRPs, should evolve more divergently in response to natural selection, primary reproductive traits can indeed experience both sexual and natural selection (e.g., Arnqvist et al., 2021; House et al., 2013). On the other hand, relaxed sexual selection on SFPs and FRPs in WSS lines could in theory generate greater divergence between WSS lines by genetic drift, especially since many SFPs and FRPs show sex-biased expression (Dapper & Wade, 2020). Seen in light of these considerations, the greater expression divergence of SFPs and FRPs relative to proteins in general in SSS lines is at least consistent with a causal role for sexual selection (Edward et al., 2010).

We found no consistent directionality in the pattern of the outcome of sperm competition across evolving lines, in the sense that within-population crosses did not generally show the highest last male paternity. At a first glance, this may seem surprising as we generally expect sexual selection to drive the coevolution of male and female traits in polygenic space, resulting in interactions between male and female population identity in the outcome of mating (Andrés & Arnqvist, 2001). However, because of the multidimensional nature of trait space, potentially high allelic diversity of genes encoding male and female traits (Gavrilets & Waxman, 2002; Haygood, 2004) and idiosyncrasies between evolving populations, the very early stage of divergence between populations is not generally expected to result in a consistent directionality in the pattern of population crosses (Andrés & Arnqvist, 2001; Long et al., 2006 ; Pitnick et al., 2009; Rowe et al., 2003; Tregenza et al., 2006). Given enough time, however, coevolution is expected to cause sufficient divergence to result in incompatibilities between populations (Mendelson et al., 2014; Price, 1997; Rundle & Rowe, 2018).

In conclusion, our findings are consistent with a scenario in which sexual selection drives the divergent evolution of reproductive traits in allopatric lineages of the seed beetle *A. obtectus*. This is not only apparent from the pattern of male–female interactions in sperm competition success, but the more divergent evolution of SFPs and FRPs under stronger sexual selection also offers at least a potential causal mechanism for this finding. In this sense, our study represents an addition to our understanding of how sexual selection can promote divergence that may ultimately lead to speciation. Yet, as in all experimental evolution studies, a role for natural selection in divergence is difficult to exclude entirely and the link between reproductive proteins and sexual selection is inferred rather than demonstrated. Future experimental evolution studies may consider using multiple experimental strategies to manipulate the strength of sexual selection, dedicated species-specific annotations of candidate genes and functional genomics to strengthen the link between sexual selection and the genetic basis of evolving reproductive phenotypes.

## Data Availability

All experimental data have been published in Mendeley Data and are publicly available at https://data.mendeley.com/datasets/d25jkfv7gv/1. Raw RNA-Seq data are deposited in FASTQ format to the NCBI Sequence Read Archive database (SRA) under the BioProject accession number PRJNA492259.
